# Magnetic vagus nerve stimulation alleviates myocardial ischemia-reperfusion injury by the inhibition of pyroptosis through the M_2_AChR/OGDHL/ROS axis in rats

**DOI:** 10.1186/s12951-023-02189-3

**Published:** 2023-11-14

**Authors:** Yao Lu, Kaiyan Chen, Wei Zhao, Yan Hua, Siyuan Bao, Jian Zhang, Tianyu Wu, Gaoyuan Ge, Yue Yu, Jianfei Sun, Fengxiang Zhang

**Affiliations:** 1 Section of Pacing and Electrophysiology, Division of Cardiology, The First Affiliated Hospital of Nanjing Medical University, Guangzhou Road 300, Nanjing, 210029 PR China; 2 Department of Cardiology, Xuzhou Central Hospital, Xuzhou Clinical School of Nanjing Medical University, No.199 Jiefang South Road, Xuzhou, 221009 PR China; 3 The State Key Laboratory of Bioelectronics, Jiangsu Key Laboratory of Biomaterials and Devices, School of Biological Science and Medical Engineering, Southeast University, Nanjing, 210009 P. R. China; 4 Department of Echocardiography, Zhongshan Hospital, Shanghai Institute of Cardiovascular Diseases, Shanghai Institute of Medical Imaging, Fudan University, 180 Fenglin Road, Shanghai, China

**Keywords:** Myocardial I/R injury, Vagus nerve, mVNS, Pyroptosis, OGDHL

## Abstract

**Background:**

Myocardial ischemia-reperfusion (I/R) injury is accompanied by an imbalance in the cardiac autonomic nervous system, characterized by over-activated sympathetic tone and reduced vagal nerve activity. In our preceding study, we pioneered the development of the magnetic vagus nerve stimulation (mVNS) system. This system showcased precise vagus nerve stimulation, demonstrating remarkable effectiveness and safety in treating myocardial infarction. However, it remains uncertain whether mVNS can mitigate myocardial I/R injury and its specific underlying mechanisms. In this study, we utilized a rat model of myocardial I/R injury to delve into the therapeutic potential of mVNS against this type of injury.

**Results:**

Our findings revealed that mVNS treatment led to a reduction in myocardial infarct size, a decrease in ventricular fibrillation (VF) incidence and a curbing of inflammatory cytokine release. Mechanistically, mVNS demonstrated beneficial effects on myocardial I/R injury by inhibiting NLRP3-mediated pyroptosis through the M_2_AChR/OGDHL/ROS axis.

**Conclusions:**

Collectively, these outcomes highlight the promising potential of mVNS as a treatment strategy for myocardial I/R injury.

**Supplementary Information:**

The online version contains supplementary material available at 10.1186/s12951-023-02189-3.

## Introduction

Reperfusion therapy for acute coronary syndromes, including surgical coronary artery bypass graft, primary percutaneous coronary intervention (PPCI) as well as thrombolytic therapy has developed rapidly in recent years [1]. However, the I/R injury caused by reperfusion therapy also induces unwanted cardiac injury, which adversely affect the prognosis of patients [2, 3]. Although advances in preventive and therapeutic measures for myocardial I/R injury have improved outcomes, but the complex clinical environment still lacks a truly effective solution [4].

Previous studies have demonstrated that myocardial I/R injury is influenced by cardiac autonomic imbalances, which manifest as a decrease in vagal activity coupled with an excessive activation of sympathetic activity [5–7]. Although many experimental and clinical studies demonstrate that electrical vagus nerve stimulation (eVNS) exhibits a beneficial impact in alleviating myocardial I/R injury via the enhancement of vagal activity [8, 9], but electrical stimulator implantation via surgery may lead to adverse outcomes such as postoperative infections, venous thrombosis, wire displacement and battery exhaustion [10]. Therefore, it is urgent to develop a new vagal modulation technique. In our previous research, we innovatively designed the mVNS system. This system comprised an injectable magnetic hydrogel infused with superparamagnetic iron oxide (SPIO) nanoparticles, which was utilized in conjunction with a gentle magnetic field. Through this approach, we were able to stimulate the cervical vagus nerve precisely and effectively in rats and then confirmed the efficacy, safety and stability of mVNS as a treatment for heart failure following myocardial infarction [11]. However, it is not clear whether mVNS can improve myocardial I/R injury, which needs further study.

Pyroptosis, a newly discovered cell death type, has been proven to be programmed by an inflammatory caspase [12]. NLRP3-mediated pyroptosis is characterized by the activation of the NLRP3 inflammasome and its subsequent release of inflammatory factors such as interleukin IL-1βand IL-18 [13, 14]. More importantly, mangy studies demonstrate that NLRP3 inflammasome-mediated pyroptosis takes part in myocardial I/R injury [13, 15]. In recent years, numerous studies have suggested that vagal stimulation could inhibit the activation of the NLRP3 inflammasome by the cholinergic anti-inflammatory pathway (CAP), which through the binding of acetylcholine (ACh) to the α7 subunit of the nicotinic acetylcholine receptor (α7nAChR) present on macrophages [16]. Although one study demonstrated that VNS exerts cardioprotection against doxorubicin-induced cardiotoxicity through inhibition of pyroptosis [17]. However, no research has analyzed the correlation between pyroptosis and VNS in myocardial I/R injury. Here, we suppose that mVNS could exert anti-pyroptosis effects following myocardial I/R injury through the mechanism of ACh binding to cardiomyocyte ACh receptors. Additionally, there are multiple types of ACh receptors on the cardiomyocyte and further investigation has been conducted to determine which receptor ACh binds to in order to exert its anti-pyroptosis effects.

Cumulative evidence has shown that VNS could alleviate reactive oxygen species (ROS) accumulation and improve mitochondrial energy metabolism under myocardial ischemia damage [18, 19]. Numerous studies have indicated that the abnormal buildup of ROS can trigger the excessive activation of NLRP3 inflammasomes, ultimately leading to pyroptosis, which has been identified as a critical factor in the development and progression of myocardial I/R injury [20, 21]. These results suggested us whether mVNS can play an anti-pyroptosis role by regulating ROS accumulation. OGDHL (2-oxoglutarate dehydrogenase like), as the rate-limiting component of the mitochondrial multi-enzyme OGDH complex (OGDHC), emerges as a pivotal enzyme within tricarboxylic acid (TCA) cycle, renowned for its ability to orchestrate the remarkable conversion of 2-oxoglutarate to succinyl-CoA [22, 23]. It has been shown that OGDHL regulated succinate metabolization and served as an important ROS generator in cardiac mitochondria [24]. Another study found that the abnormal accumulation of succinate leads to the excessive mitochondrial reactive oxygen species (mtROS) production in the case of I/R injury [25]. Hence, we hypothesize that the overexpression of OGDHL leads to abnormal succinate accumulation and then excessive mtROS production in myocardial I/R injury. While mVNS can alleviate excessive mtROS production by inhibiting the overexpression of OGDHL and then lead to the amelioration of myocardial pyroptosis, ultimately achieving a therapeutic effect.

In pursuit of these goals, our research sought to provide a deeper grasp of the mechanisms underlying mVNS-mediated cardioprotection and its potential in alleviating the adverse effects of myocardial I/R injury. We centered on investigating whether the implementation of mVNS could reduce OGDHL overexpression, consequently resulting in a reduction in mtROS production and a suppression of myocardial pyroptosis.

## Results

### Establishment of the magnetic stimulation system and magnetic vagus nerve stimulation

**Fig. 1:**
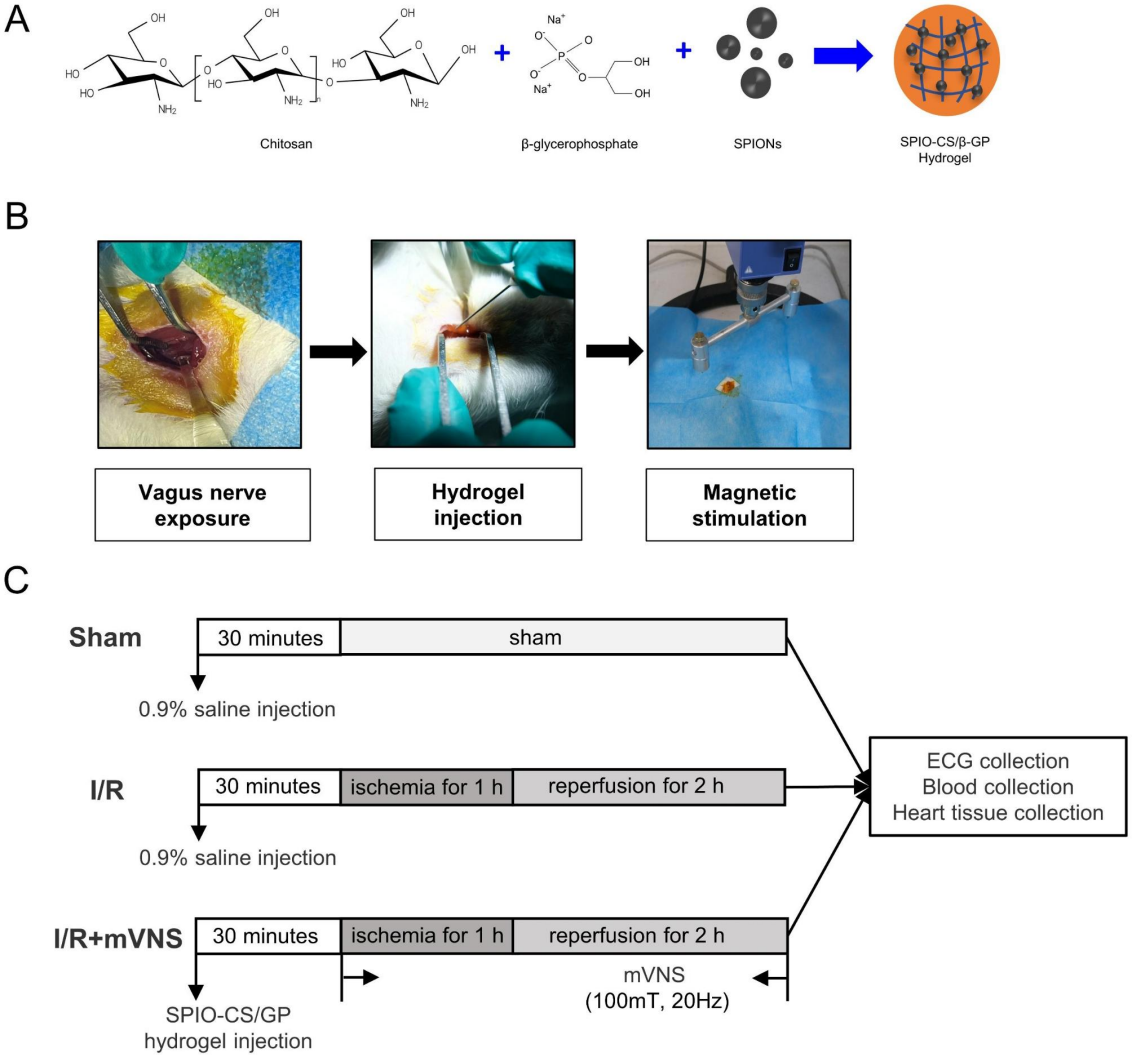
Establishment of the magnetic stimulation system and magnetic vagus nerve stimulation. (**A**) The synthetic way of SPIO-CS/GP hydrogel. (**B**) SPIO-CS/GP hydrogel injection and magnetic vagus nerve stimulation. (**C**) Schematic representation of mVNS treatment for myocardial ischemia-reperfusion injury

In this study, with a 9 nm iron oxide core and a glycosides coating layer over 20 nm, SPIO nanoparticles approved by FDA for clinical use combine more effectively with chitosan hydrogel. We successfully synthesized the SPIO-CS/GP temperature-sensitive hydrogel (Fig. 1A). During the animal experiments, we firstly separated the right vagus nerve of SD rats and then coated the SPIO-CS/GP hydrogel onto the vagus nerve surface and stimulate the right vagus nerve magnetically once the cone magnet passed by (Fig. 1B). Figure 1C exhibited the schematic representation of mVNS treatment for myocardial I/R injury (Fig. 1C).

**Fig. 2:**
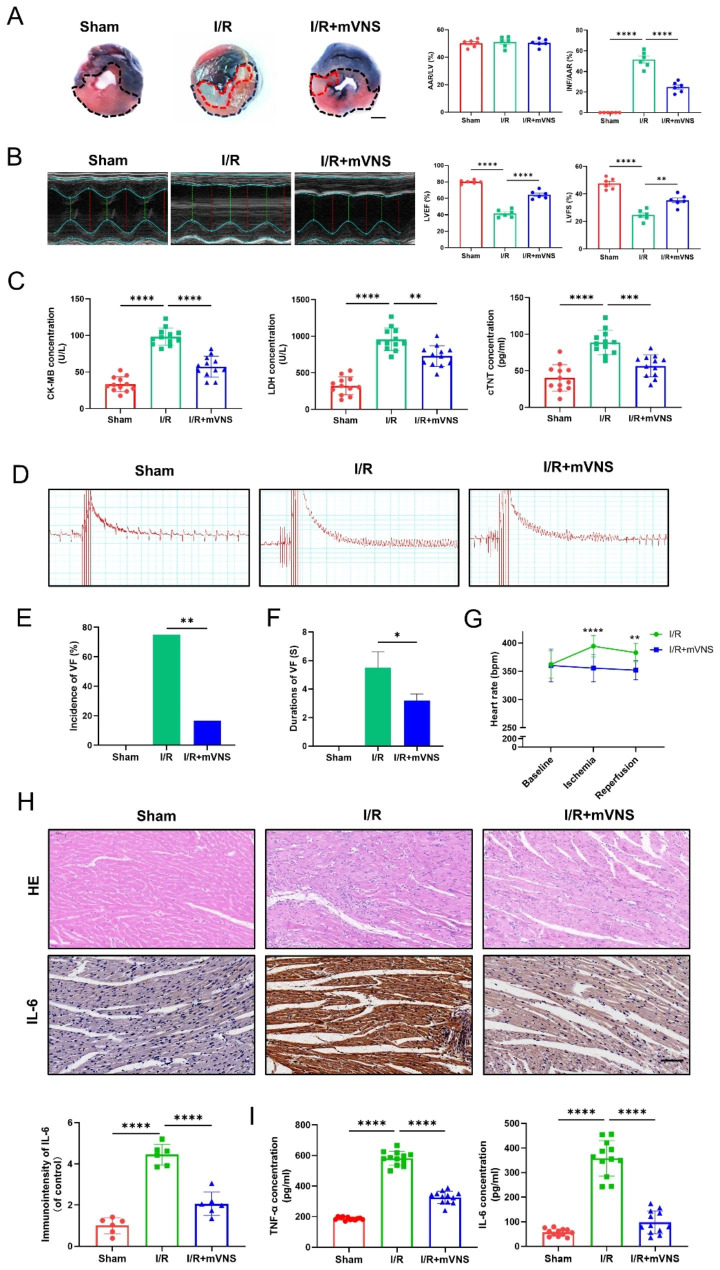
mVNS treatment attenuated myocardial I/R injury, ventricular fibrillation and inflammatory cytokine release. (**A**) Evans blue and TTC staining was used to detect the infarct size of I/R rats. The area of normally supplied blood was blue, the area at risk (AAR) was red and that of infarct size (INF) was pale. INF/AAR reflects the level of dead myocardium, n = 6 per group, Scale bar: 3 mm. (**B**) Representative echocardiographic images showed heart function 24 h after I/R and quantitative analysis of left ventricular ejection fraction (LVEF) and left ventricular fraction shortening (LVFS) among the different groups, n = 6 per group. (**C**) Effects of mVNS treatment on the myocardial enzyme concentrations in serum by Elisa, n = 12 per group. (**D**) Representative electrocardiographic tracings illustrating the inhibitory effect of mVNS on I/R-induced VF. (**E**) Effect of mVNS on the incidence of VF, n = 12 per group. (**F**) Effect of mVNS on VF duration, n = 12 per group. (**G**) Effect of mVNS on the heart rate, n = 12 per group. (**H**) mVNS treatment decreased inflammatory cell infiltration and IL-6 expression in myocardial tissue, n = 6 per group. Scale bar: 100 μm. (**I**) Measurements of mVNS treatment on the concentration of TNF-α and IL-6 in serum by Elisa, n = 12 per group. Data are mean ± SEM. *P < 0.05, **P < 0.01, ***P < 0.001, ****P < 0.0001; NS means not significant between groups. All P values were obtained by one-way ANOVA or Chi square test

### mVNS treatment attenuated myocardial I/R injury, ventricular fibrillation and inflammatory cytokine release

Compared to I/R group, mVNS apparently reduced the infarct size in the heart and improved cardiac function (Fig. 2A and B), while the increased myocardial enzymes concentration in serum induced by I/R injury was significantly decreased with mVNS treatment (Fig. 2C). The antiarrhythmic effect of mVNS treatment was assessed using the I/R-induced ventricular arrhythmia model with ischemia for 1 h followed by reperfusion for 2 h. At the end of reperfusion, electrocardiogram data were collected after programmed electrical stimulation (Fig. 2D). We found that mVNS could significantly reduce the incidence and duration of I/R-induced VF (Fig. 2E, F). The results also showed that I/R injury significantly increased the heart rate compared with sham group, while mVNS obviously reduced higher heart rate induced by I/R injury (Fig. 2G). Moreover, compared with the sham group, the inflammatory cell infiltration and IL-6 expression in the myocardium of I/R rats were significantly increased. However, mVNS treatment alleviated the inflammatory cell infiltration and expression of IL-6 in myocardium (Fig. 2H). Similar changes can also be seen in the expression of IL-6 and TNF-α in serum. mVNS could reverse the I/R-induced increasement of IL-6 and TNF-α concentration in serum (Fig. 2I).

### mVNS reduced myocardium pyroptosis induced by the myocardial I/R injury

**Fig. 3:**
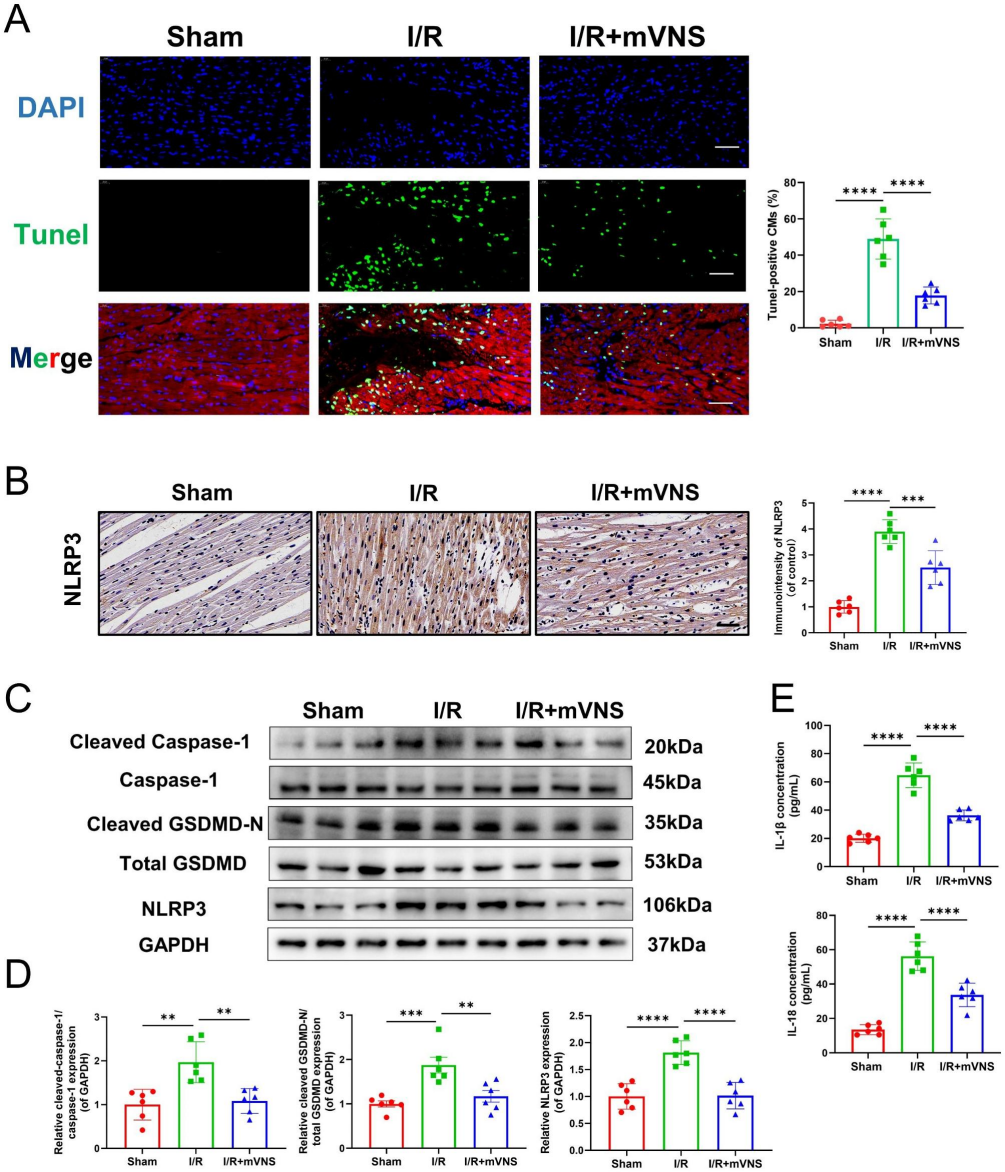
mVNS reduced myocardium pyroptosis induced by the myocardial I/R injury. (**A**) TUNEL analysis for effect of mVNS on myocardial pyroptosis under I/R injury. Green, TUNEL-positive nuclei; blue, DAPI-stained nuclei myocardium, n = 6 per group. Scale bar: 50 μm. (**B**) Immunohistochemical observation of NLRP3-positive expression under mVNS treatment, n = 6 per group. Scale bar: 50 μm. (**C**) Representative western blot of NLRP3, Cleaved Caspase-1, Caspase-1, Total GSDMD and Cleaved GSDMD-N in myocardium of rats after I/R and mVNS treatment, n = 6 per group, GAPDH as an internal control. (**D**) Protein quantification based on the results of the 3 C. (**E**) ELISA was used to measure the levels of IL-1β and IL-18 in myocardium of rats after I/R and mVNS treatment, n = 6 per group. Data are mean ± SEM. *P < 0.05, **P < 0.01, ***P < 0.001, ****P < 0.0001; NS means not significant between groups. All P values were obtained by one-way ANOVA

We measured the Tunel positive cardiomyocytes in myocardium. The result indicated that compared to I/R group, mVSN treatment effectively relieved Tunel positive cardiomyocytes (Fig. 3A). Subsequently, we assessed the pyroptosis indicators within the myocardial tissue of rats subjected to I/R injury. Notably, compared with sham group, there was a substantial rise in the myocardial expression of NLRP3 in I/R group (Fig. 3B-D). Cleaved caspase-1 and cleaved N-terminal GSDMD expression in myocardium were elevated (Fig. 3C and D) and a significant quantity of inflammatory factors (IL-1β and IL-18) were released in I/R group (Fig. 3E). Nevertheless, the levels of pyroptosis-related proteins within the myocardial tissue and the release of inflammatory factors were alleviated after mVNS treatment (Fig. 3B-E). It is suggested that mVNS exhibited the ability to inhibit pyroptosis induced by I/R.

### mVNS regulated myocardial pyroptosis by suppressing OGDHL expression and improving mitochondrial damage in vivo

**Fig. 4:**
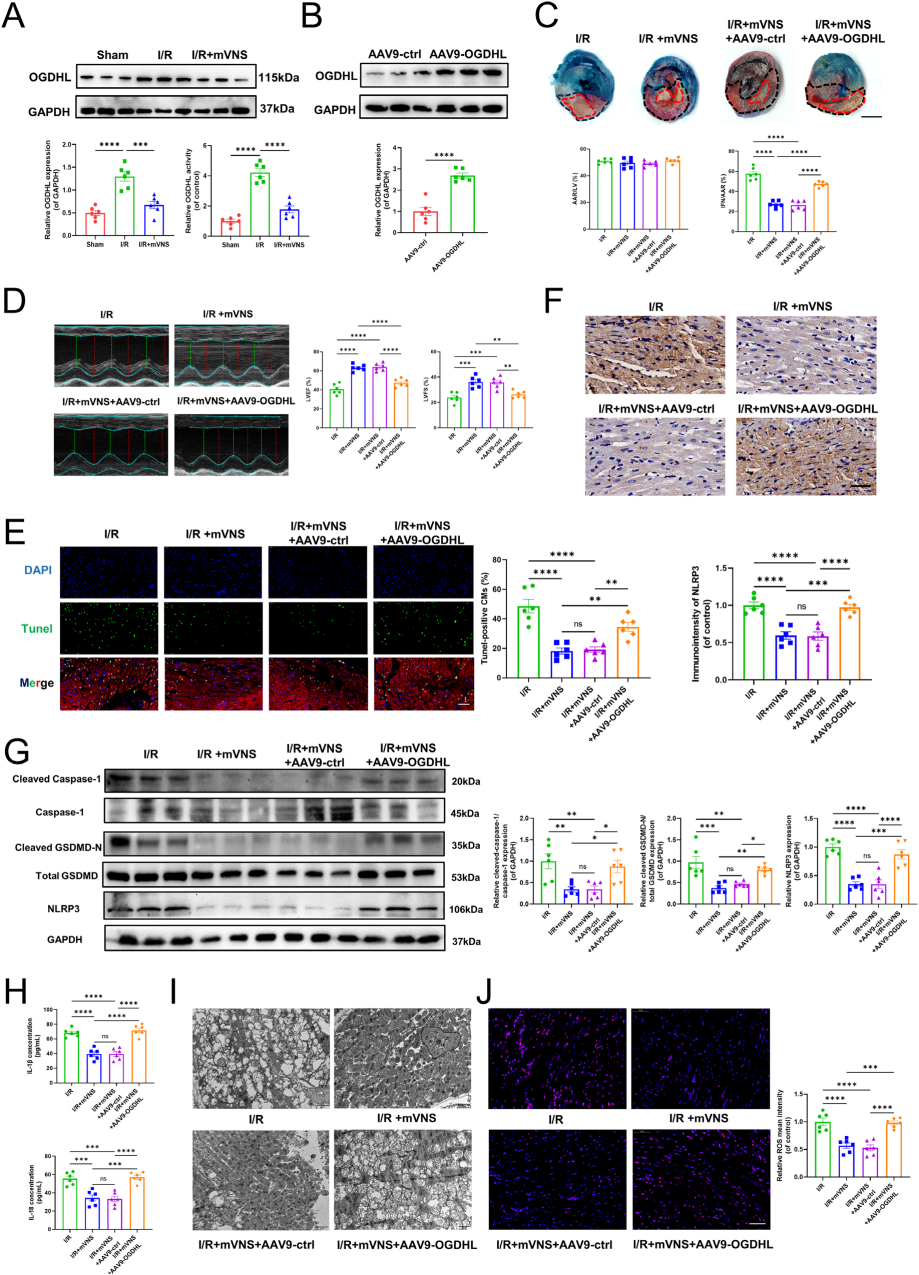
mVNS regulated myocardial pyroptosis by suppressing OGDHL expression and improving mitochondrial damage in vivo. (**A**) Western blot analysis of protein level of OGDHL (GAPDH as an internal control) and quantification of OGDHL activity in the myocardium of rats after I/R or mVNS treatment, n = 6 per group. (**B**) Western blot analysis of OGDHL expression in heart tissue transfected OGDHL, n = 6 per group. GAPDH as an internal control. (**C**-**J**) Rats were transfected with AAV9-ctrl or AAV9-cTNT-OGDHL three week before I/R surgery and mVNS tratment. (**C**) Evans blue and TTC staining was used to detect the infarct size of I/R rats. The area of normally supplied blood was blue, the AAR was red and that of INF was pale. INF/AAR reflects the level of dead myocardium., n = 6 per group, Scale bar: 3 mm. (**D**) Representative echocardiographic images showed heart function 24 h after I/R and quantitative analysis of LVEF and LVFS among the different groups, n = 6 per group. (**E**) TUNEL analysis for myocardial pyroptosis. Green, TUNEL-positive nuclei; blue, DAPI-stained nuclei, n = 6 per group. Scale bar: 50 μm. (**F**) Immunohistochemical observation of NLRP3-positive expression, n = 6 per group. Scale bar: 40 μm. (**G**) Western blot analysis of protein levels of NLRP3, Cleaved Caspase-1, Caspase-1, Total GSDMD and Cleaved GSDMD-N in myocardium and quantification, n = 6 per group, GAPDH as an internal control. (**H**) ELISA was used to measure the levels of IL-1β and IL-18 in myocardium, n = 6 per group. (**I**) Representative images of mitochondrial ultrastructure of myocardium with TEM, n = 6 per group. Scale bar: 2 μm. (**J**) Representative images of mtROS production in myocardium and quantification of relative mtROS mean intensity, n = 6 per group. Scale bar: 50 μm. Data are mean ± SEM. *P < 0.05, **P < 0.01, ***P < 0.001, ****P < 0.0001; NS means not significant between groups. All P values were obtained by one-way ANOVA

We initially confirmed that OGDHL expression and activity were significantly elevated under myocardial I/R injury, while mVNS can reverse the upregulation of OGDHL expression and activity (Fig. 4A). To verify the role of OGDHL in mVNS alleviating myocardial pyroptosis, AAV9-cTNT-OGDHL was used to overexpress OGDHL expression in myocardium. Western blot showed that the expression of OGDHL was obviously elevated in myocardium by virus transfection (Fig. 4B). Evans Blue/TTC double staining revealed that OGDHL overexpression attenuated the effect of mVNS in reducing myocardial infarction size (Fig. 4C). Echocardiography indicates that OGDHL overexpression diminishes the improvement of cardiac function by mVNS (Fig. 4D). Similarly, raising the expression level of OGDHL hindered the ability of mVNS to alleviate myocardium pyroptosis and inflammatory cytokines release (Fig. 4F-H). We further examined the mitochondria in myocardial tissue and observed that mVNS significantly improved mitochondrial structural damage and mtROS production caused by myocardial I/R injury. However, OGDHL overexpression hindered the mVNS-mediated improvement of mitochondrial damage and mtROS production in myocardium (Fig. 4I, J).

### ACh reduced pyroptosis by reversing elevated OGDHL expression and mitigating mitochondrial damage in NRCMs

**Fig. 5:**
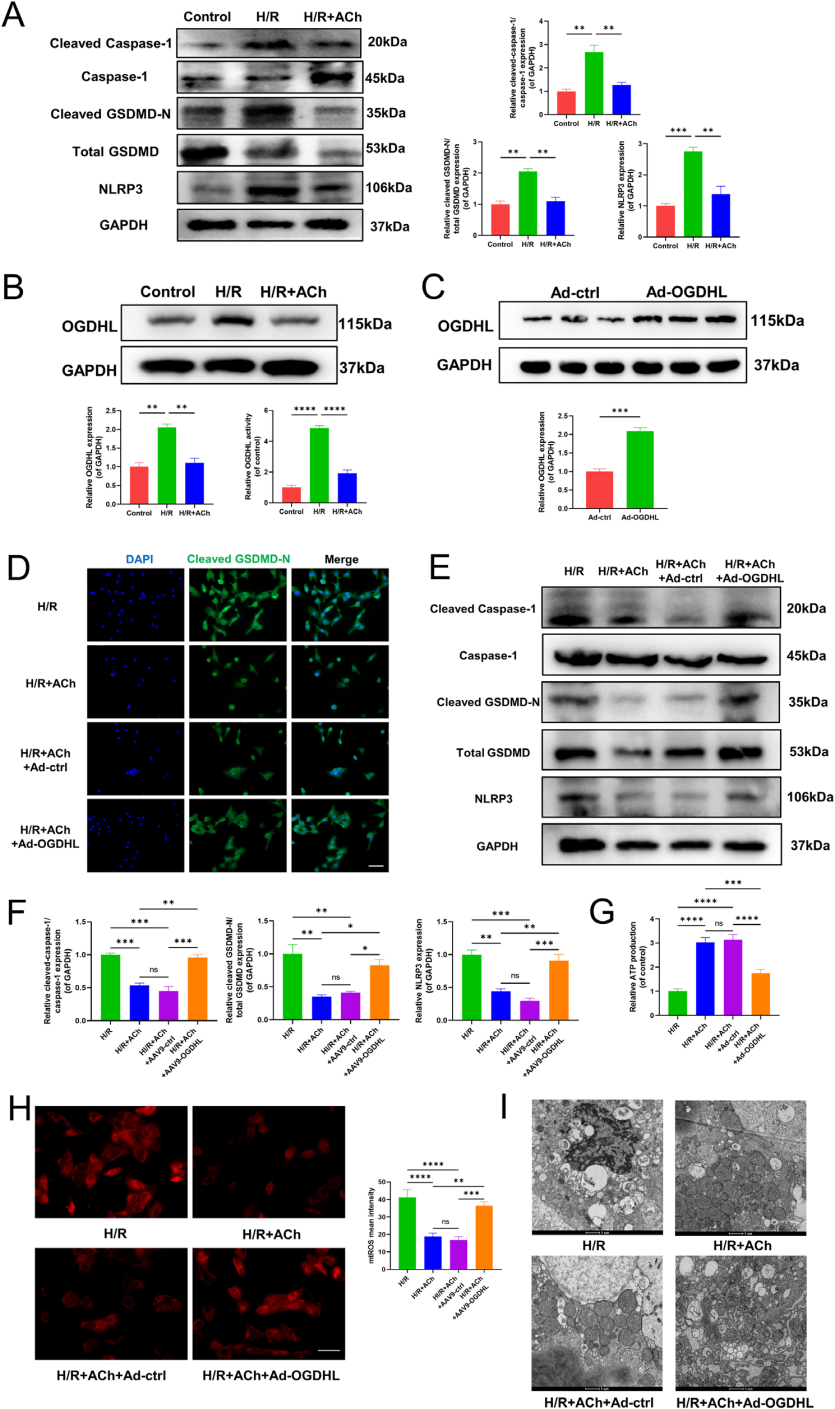
ACh reduced pyroptosis by reversing elevated OGDHL expression and mitigating mitochondrial damage in NRCMs. (**A**) Western blot analysis of protein levels of NLRP3, Cleaved Caspase-1, Caspase-1, Total GSDMD and Cleaved GSDMD-N in NRCMs after H/R or ACh treatment and quantification, n = 3 per group, GAPDH as an internal control. (**B**) Western blot analysis of protein level of OGDHL (GAPDH as an internal control) and quantification of OGDHL activity in NRCMs after H/R or ACh treatment, n = 3 per group. (**C**-**I**) NRCMs were transfected with Ad-ctrl or Ad-OGDHL before H/R and ACh treatment. (**C**) Western blot analysis of OGDHL expression in NRCMs transfected OGDHL, n = 3 per group. GAPDH as an internal control. (**D**) Immunofluorescence staining of NRCMs for GSDMD-N (green) and DAPI (blue). Translocation showed membrane or cytoplasmic localization of GSDMD-N, Scale bar: 100 μm. (**E**) Western blot analysis of protein levels of NLRP3, Cleaved Caspase-1, Caspase-1, Total GSDMD and Cleaved GSDMD-N in NRCMs, n = 3 per group, GAPDH as an internal control. (**F**) Protein quantification based on the results of the 3E. (**G**) Relative ATP production of in NRCMs, n = 3 per group. (**H**) Representative images of mtROS production in NRCMs and quantification of relative mtROS mean intensity, n = 6 per group, Scale bar: 100 μm. (**I**) Representative images of mitochondrial ultrastructure of myocardium with TEM, n = 6 per group. Scale bar: 1 μm. Data are mean ± SEM. *P < 0.05, **P < 0.01, ***P < 0.001, ****P < 0.0001; NS means not significant between groups. All P values were obtained by one-way ANOVA

Firstly, we confirmed the effectiveness of the in vitro H/R model in inducing pyroptosis and the effectiveness of ACh (Fig. 5A). We validated the raising of OGDHL expression and activity by OGD and reoxygenation, while ACh was found to reverse the upregulation of OGDHL and activity in NRCMs (Fig. 5B). To confirm the involvement of OGDHL in the mitigating effects of ACh on pyroptosis, Ad-OGDHL was employed to upregulate OGDHL expression in NRCMs. Western blot analysis revealed a noticeable increasement in OGDHL expression in NRCMs following Ad-OGDHL transfection (Fig. 5C). As shown in Fig. 5D, ACh treatment reversed the translocation of cleaved GSDMD-N from cytosol to plasma membrane in the presence of H/R, while OGDHL overexpression weakened the effect of ACh on cleaved GSDMD-N translocation (Fig. 5D). Moreover, our study revealed that the OGDHL overexpression in NRCMs inhibited the benefic effects of ACh in alleviating pyroptosis-related proteins expression (Fig. 5E, F). Furthermore, we assessed mtROS and mitochondrial function in NRCMs. The results indicated that OGD and reoxygenation led to a substantial generation of mtROS and significant mitochondrial damage, and ACh treatment suppressed ROS production, improved mitochondrial structure, and enhanced ATP production. While OGDHL overexpression hindered the ACh-mediated improvement of mitochondrial damage and mtROS production in NRCMs (Fig. 5G-I).

### mVNS activates M_2_AChR to suppress OGDHL expression and pyroptosis in vivo

**Fig. 6:**
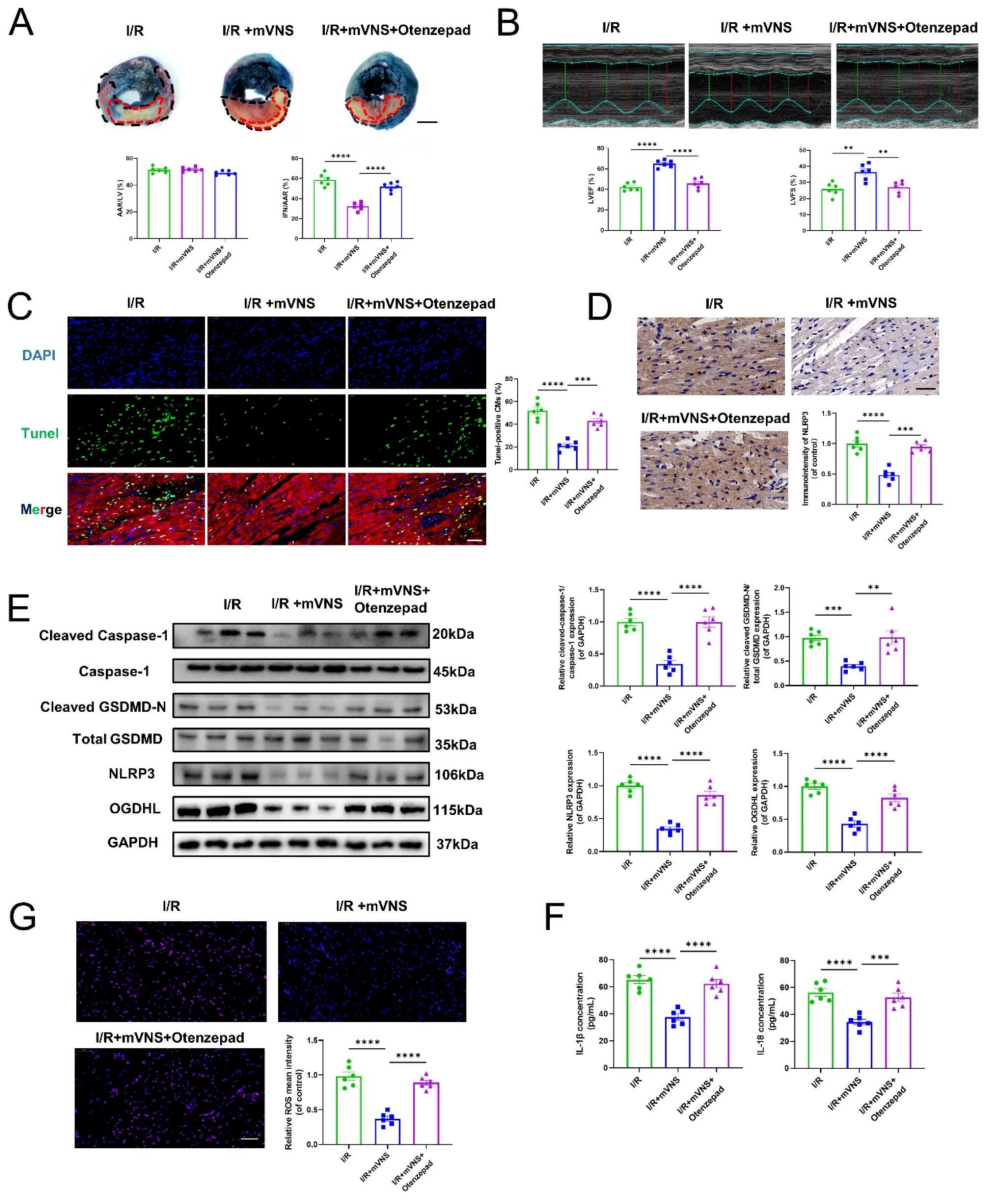
mVNS activates M_2_AChR to suppress OGDHL expression and pyroptosis in vivo. (**A**) Evans blue and TTC staining was used to detect the infarct size of I/R rats. The area of normally supplied blood was blue, the AAR was red and that of INF was pale. INF/AAR reflects the level of dead myocardium., n = 6 per group, Scale bar: 3 mm. (**B**) Representative echocardiographic images showed heart function 24 h after I/R and quantitative analysis of LVEF and LVFS among the different groups, n = 6 per group. (**C**) TUNEL analysis for myocardial pyroptosis. Green, TUNEL-positive nuclei; blue, DAPI-stained nuclei, n = 6 per group. Scale bar: 50 μm. (**D**) Immunohistochemical observation of NLRP3-positive expression, n = 6 per group. Scale bar: 40 μm. (**E**) Western blot analysis of protein levels of OGDHL, NLRP3, Cleaved Caspase-1, Caspase-1, Total GSDMD and Cleaved GSDMD-N in myocardium and quantification, n = 6 per group, GAPDH as an internal control. (**F**) ELISA was used to measure the levels of IL-1β and IL-18 in myocardium, n = 6 per group. (**G**) Representative images of mtROS production in myocardium and quantification of relative mtROS mean intensity, n = 6 per group. Scale bar: 50 μm. Data are mean ± SEM. *P < 0.05, **P < 0.01, ***P < 0.001, ****P < 0.0001; NS means not significant between groups. All P values were obtained by one-way ANOVA

To further investigate whether M_2_AChR is involved in the anti-pyroptotic effect of mVNS, we utilized the M_2_AChR antagonist Otenzepad to inhibit the activation of M_2_AChR. The results showed that M_2_AChR antagonist Otenzepad attenuated the effects of mVNS in reducing myocardial infarction size and improving cardiac function (Fig. 6A and B). Further investigations revealed that the anti-pyroptotic effects of mVNS were also suppressed by inhibiting the activity of M_2_AChR (Fig. 6D-F). Similarly, we also observed that M_2_AChR antagonist Otenzepad promoted OGDHL expression, subsequently leading to increased ROS production in heart tissue (Fig. 6E and G).

### ACh activates M_2_AChR to inhibit OGDHL expression and activity to counteract pyroptosis in NRCMs

**Fig. 7:**
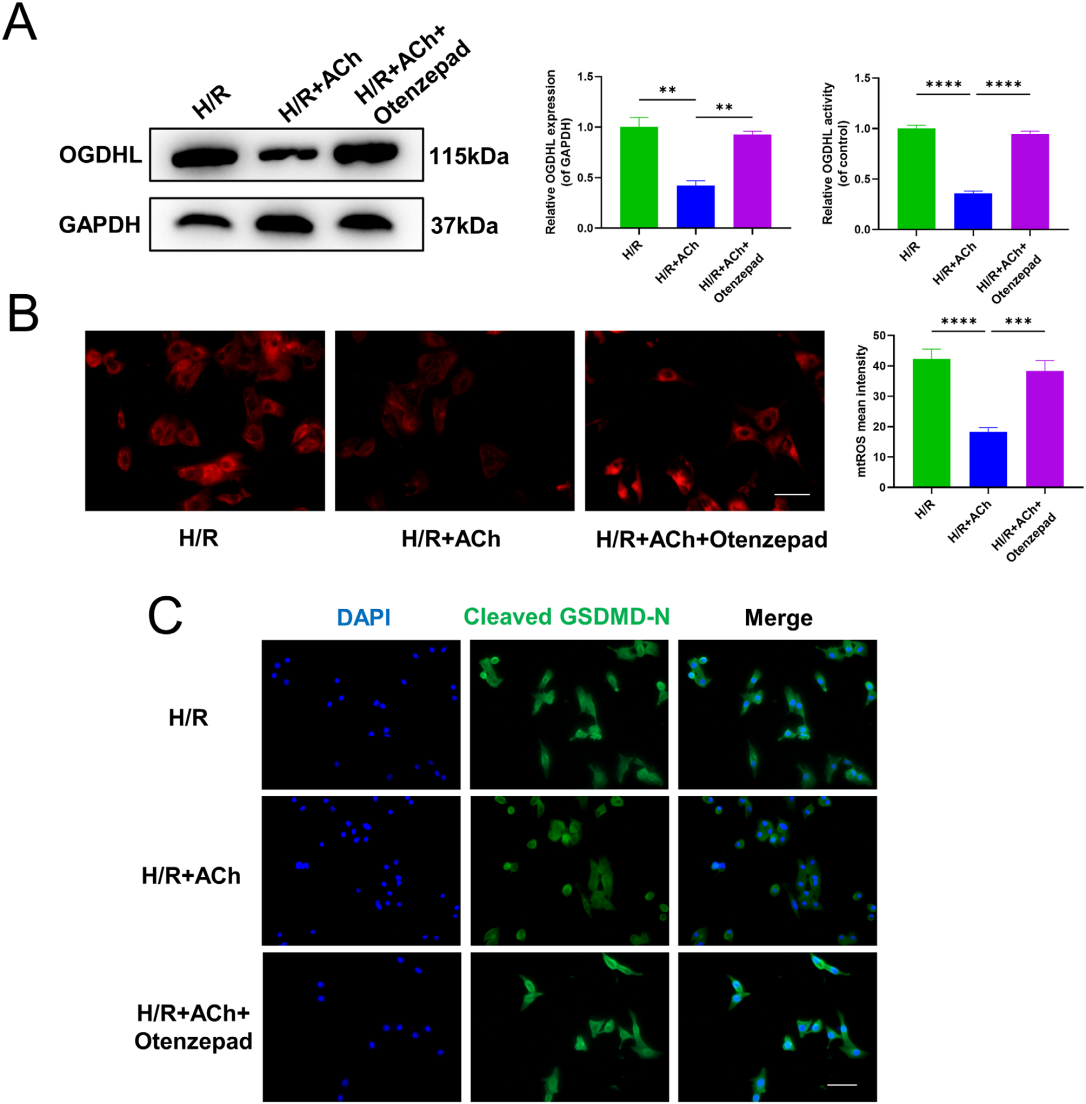
ACh activates M_2_AChR to inhibit OGDHL expression and activity to counteract pyroptosis in NRCMs. NRCMs was incubated with or without Otenzepad (an M_2_ receptor antagonist) under H/R or ACh treatment. (**A**) Western blot analysis of protein level of OGDHL (GAPDH as an internal control) and quantification of OGDHL activity in NRCMs after H/R or ACh treatment, n = 3 per group. (**B**) Representative images of mtROS production in NRCMs and quantification of mtROS mean intensity, n = 6 per group, Scale bar: 100 μm. (**C**) Immunofluorescence staining of NRCMs for GSDMD-N (green) and DAPI (blue). Translocation showed membrane or cytoplasmic localization of GSDMD-N, Scale bar: 100 μm

We also assessed the impact of M_2_AChR antagonist Otenzepad on the inhibitory effect of ACh in vitro. The results revealed that the Otenzepad weakened the impact of ACh on suppression OGDHL expression levels (Fig. 7A). mtROS fluorescence staining demonstrated that the M_2_AChR antagonist Otenzepad diminished the effects of ACh in inhibiting excessive mtROS generation (Fig. 7B). As anticipated, the M_2_AChR antagonist Otenzepad also inhibited the effect of ACh in suppression cleaved GSDMD-N translocation in NRCMs (Fig. 7C).

## Discussion

**Fig. 8:**
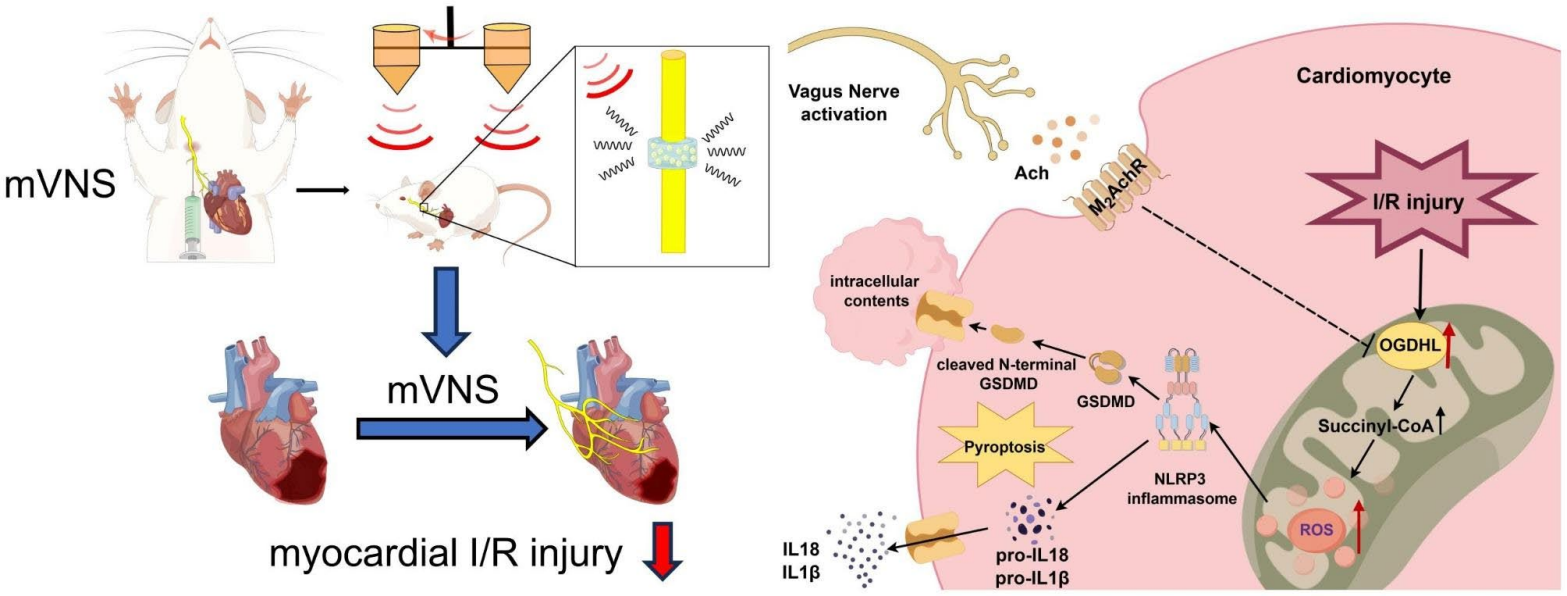
Inhibitory effect of mVNS on pyroptosis induced by myocardial I/R injury. Schematic diagram showing the potential molecular mechanisms through which mVNS alleviates myocardial I/R injury by inhibiting NLRP3-mediated pyroptosis through the M_2_AChR/OGDHL/ROS axis

In this study, we introduced a novel approach by employing a mVNS system based on the SPIO-CS/GP hydrogel in conjunction with a magnetic field to alleviate myocardial I/R injury. mVNS treatment exhibited the capacity to notably reduce myocardial damage and the occurrence of ventricular arrhythmias. Furthermore, our findings revealed that mVNS exerted a cardioprotective impact on myocardial I/R injury by effectively suppressing pyroptosis through modulation of the M_2_AChR/OGDHL/ROS axis (Fig. 8). These results collectively highlight the therapeutic potential of mVNS in the context of myocardial I/R injury and provide novel insights into the mechanisms underlying the actions of mVNS.

Cardiac autonomic imbalance has been linked to elevated mortality rates among patients with myocardial I/R injury [5]. Previous studies have demonstrated that over-activation of the sympathetic nervous system could further promote the myocardial inflammatory reactions, the extent of infarct size and arrhythmic episodes [26–28]. Therefore, restoring parasympathetic nerve activity is essential to ameliorate myocardial I/R injury.

The vagus nerve is an important peripheral nerve member of the parasympathetic autonomic nervous system and a key nerve in the reflex pathway that regulates cardiac function [29]. Within this study, we confirmed the therapeutic benefits of mVNS as an alternative to eVNS due to its ability to circumvent certain side effects, all while mitigating myocardial I/R injury. Our findings revealed that mVNS treatment resulted in a reduction in myocardial infarct size, a decrease in ventricular fibrillation (VF) incidence, and a curbing of inflammatory cytokine release. During the treatment, we choose 20 Hz as the magnetic stimulation frequency considering the FDA’s approval for electrical stimulation frequency and the results turns out that this strategy offers great cardiac protection against myocardial I/R injury.

Ventricular arrhythmias (VAs), which is regarded as one of the major complications induced by myocardial I/R injury, has a high risk of sudden cardiac death [30]. A series of studies have found that VNS could reduce the incidence of severe VAs in acute myocardial I/R injury and this protective effect of VNS could be abolished by vagotomy and atropine [19, 31]. Our study also found that mVNS can exert cardioprotective effect by reducing the incidence and duration of VF induced by myocardial I/R injury. Previous researches have indicated that lower heart rate might have a higher threshold for VAs [32, 33]. Therefore, reduced heart rate may be one of the anti-arrhythmic mechanisms of mVNS.

Significant local and systemic inflammation represent significant pathophysiological mechanisms implicated in the context of myocardial I/R injury [34]. The vagal nerve is recognized as a significant constituent of the neuroendocrine-immune axis. Lieder et al. first reported that vago-splenic axis immediately participated in acute myocardial I/R injury with a beneficial effect on inflammatory cytokine and infarct size [35]. In our study, we also found that mVNS therapy could effectively reduce the infarct size, accompany with reducing inflammatory cytokines secretion and the infiltration of neutrophils in myocardium. Moreover, another study shown that inflammatory cytokines generated by cardiac macrophage contribute to the ventricular electrophysiology, leading to VAs development [36]. Therefore, in accordance with our findings, it can be inferred that mVNS might potentially exert an indirect anti-arrhythmic effect by dampening the expression of inflammatory cytokines.

Currently, there is accumulating evidence indicates the importance of NLRP3 inflammasome activation and pyroptosis in the pathophysiology of myocardial I/R injury [37, 38]. Previous studies found that NLRP3 inflammasome activation plays a key role in sterile inflammation, and the IL-1β induced by NLRP3 is considered as a prominent mediator for inflammation in myocardial I/R injury [39, 40]. However, more evidence suggested that mechanism by which the NLRP3 inflammasome induces myocardial infarction expansion and cardiomyocyte death may be through pyroptosis rather than IL-1β production [41, 42]. A few studies suggested that VNS presented protective effects through inhibiting pyroptosis in many diseases including ARDS, cerebral I/R injury and doxorubicin-induced cardiotoxicity [17, 43, 44]. Nevertheless, no research has analyzed the correlation between pyroptosis and VNS in myocardial I/R injury. Results of our study firstly indicate that mVNS could attenuate NLRP3 inflammasome activation and pyroptosis during the myocardial I/R injury.

Extensive research has revealed the remarkable potential of stimulating the vagus nerve to reduce inflammation through CAP [8, 16]. But the exact mechanisms through which it counteracts pyroptosis in myocardial I/R injury are still not fully understood. The activation of NLRP3 in I/R injury is regulated by multiple stimuli, with mtROS being the main driver of NLRP3 activation in myocardial I/R injury [20, 42]. A prior investigation demonstrated that the selective buildup of the TCA cycle intermediate succinate serves as a widespread metabolic hallmark of ischemia in a range of tissue and plays a crucial role in controlling I/R injury by regulating mtROS levels [25]. OGDHL, which facilitates the transformation of 2-oxoglutarate into succinyl-CoA through catalysis, has been shown as an important ROS generator in cardiac mitochondria [24]. In our investigation, through the analysis of OGDHL expression and activity in cardiac tissue from rats subjected to I/R injury, as well as in NRCMs exposed to OGD and reoxygenation, we corroborated the increasement in OGDHL expression and activity under I/R injury conditions. Additionally, our observations indicated that mVNS treatment had a significant influence on reducing the expression and activity of OGDHL, leading to a decrease in mtROS production. Consequently, this cascade of events effectively hindered the activation of the NLRP3 inflammasome, subsequently impeding the process of pyroptosis.

ACh, a cornerstone of the cholinergic system (ChS), plays a pivotal role in activating the vagus nerve through an intricate array of receptors, encompassing nicotinic receptors (nAChRs) and muscarinic receptors (mAChRs) [45, 46]. Among nAChRs, α7-nAChR, which is predominantly present on immune cells and neuronal ganglia, stands out as a critical protein in the CAP [47, 48]. Meanwhile, the mAChRs, a family of G protein-coupled receptors (GPCRs), encompass a total of five distinct subtypes. Three subtypes (M1, M3, and M5) serve as excitatory facilitators, whereas the other two (M_2_ and M_4_) operate as inhibitory regulators within the ChS [45, 46]. To elucidate the precise efferent signaling pathways of remarkable anti-pyroptosis effects of mVNS during myocardial I/R injury, we used Carbachol, a class of ACh analogs known for their ability to activate both nicotinic receptors (nAChRs) and muscarinic receptors (mAChRs), to replicate the actions of mVNS. Our findings revealed that Carbachol stimulation during OGD and reoxygenation treatment exhibited a significant pyroptosis inhibition in NRCMs. The M_2_ACh receptor (M_2_AChR), which stands out as the predominant isoform in cardiac tissues, serves as an inhibitory receptor to reduce heart rate and conduction velocity [49]. Prior researches have suggested that M_2_AChR activation may ameliorate myocardial I/R injury by inhibiting ER stress-induced cell apoptosis [50] and reduce doxorubicin-induced cardiotoxicity by reducing oxidative stress and improving mitochondrial function [51]. However, it is not clear whether M_2_AChR is involved in the improvement of cardiomyocyte pyroptosis by ACh under myocardial I/R injury. In this study, by utilizing the M_2_AChR antagonist Otenzepad, we have effectively confirmed that inhibiting M_2_AChR diminishes the protective effects of ACh on suppression OGDHL expression and the consequent inhibition of pyroptosis in vivo and vitro. All findings consistently demonstrate that ACh exerts its anti-pyroptotic effect by activating the M_2_AChR/OGDHL/ROS axis, which in turn inhibits the activation of the NLRP3/caspase-1 pathway-mediated pyroptosis.

There are some limitations in this study. Firstly, the precise mechanisms by which M_2_AChR activation diminished OGDHL expression require further investigation. Secondly, the existing literature suggests that different stimulation parameters and durations can yield varying outcomes [52]. While our study demonstrated the impressive cardioprotective effects of the mVNS approach against myocardial I/R injury, the optimal therapeutic parameters for mVNS technology still require more extensive investigation.

## Conclusions

In summary, our study elucidates that mVNS treatment alleviates myocardial I/R injury through the inhibition of the pyroptosis via the M_2_AChR/OGDHL/ROS axis. As a result, our findings highlight the promising therapeutic potential of mVNS on myocardial I/R injury with great clinical translational value.

## Methods

### Animals and myocardial I/R model

Sprague-Dawley (SD) rats (Male, 200–220 g weight) were obtained from the experimental animal center of Nanjing Medical University. All animal study protocols were approved by the Ethics Committee of Nanjing Medical University and conducted in accordance with the Guide for the Care and Use of Laboratory Animals. Rats were anesthetized by intraperitoneal injection of pentobarbital sodium and the electrocardiogram (ECG) was recorded. Left thoracotomy was performed after rats were connected to ventilators, and the heart was exposed. The left anterior descending (LAD) was ligated at approximately 2 mm distal to its origin with 6 − 0 surgical sutures to achieve myocardial ischemia injury for 1 h, followed by reperfusion for 2 h. ST-segment elevation and color change of the myocardial ischemic area were observed to confirm the realization of ischemia. Apex and anterior wall of the left ventricle turned red and the ECG showed a normal ST, indicating successful perfusion.

### SPIO-CS/GP hydrogel injection and magnetic vagus nerve stimulation

A midline cervical incision was performed and the anterior cervical muscles were bluntly separated until the right vagus nerve was fully exposed. A 0.2 ml volume of hydrogel was injected around the vagus nerve using a syringe, and the hydrogel solidified and completely wrapped the nerve after 5 min.

SD rats were randomly divided into 3 groups (n = 12/group): sham group, IR group, and IR + magnetic vagus nerve stimulation (mVNS) group. Vehicle (0.9% normal saline solution) was injected around the right vagus nerve without LAD coronary artery obstruction in the sham group. The IR group experienced vehicle administration around the right vagus nerve and myocardial I/R injury. mVNS group received SPIO-CS/GP hydrogel administration and the magnetic field (100mT, 20 Hz) remained active during the whole ischemia and reperfusion time.

### Infarct size determination

At the end of reperfusion, the left anterior descending branch was slagged again. The jugular vein was separated and injected with 0.4% Evans blue dye (Sigma–Aldrich, St. Louis, MO, USA). After the heart and systemic veins turned blue, the hearts were collected and placed in heart matrixes, freezing for 20 min at -80 °C. The ventricular tissue was sliced into 1–2 mm transections in thickness, then subjected to incubation with 1% 2, 3, 5-triphenyltetrazolium chloride (TTC) (Sigma–Aldrich, St. Louis, MO, USA) for a duration of 20 min at 37 °C. Subsequent to this process, all the transections were photographed and the digital images were analyzed using Image J. The area of normally supplied blood was blue, the area at risk (AAR) was red and that of infarct size (INF) was pale. INF/AAR reflects the level of dead myocardium.

### Cardiac function assessment

Cardiac function assessment was conducted using echocardiography 24 h after I/R with the VEVO 2000 high-resolution micro-imaging system. A specialized transducer was employed to obtain measurements in the M-mode of the left ventricular short-axis. The Vevo2000 workstation software was used for the calculation of the left ventricular ejection fraction (LVEF) and left ventricular shortening rate (LVFS).

### Programmed electrical stimulation

Using an electronic stimulator (PowerLab, AD Instruments), programmed electrical stimulation (PES) was executed with one drive trains consisting of 5 stimuli (S1, 5 V, 1-ms pulse width, and 100 ms intervals), followed by a sequence of 3 additional extrastimuli (S2, S3, and S4, 5 V and 1-ms pulse width) at a cycle length (CL) of 60 ms and decreasing by 5 ms decrement to a CL of 40 ms. Ventricular-effective-refractory-period (VERP) was evaluated through S1-S2 pacing at a CL of 100 ms. Ventricular fibrillation (VF) is defined as a loss of synchronicity of the ECG plus decreased amplitude with heart rate > 250 beats/minute according to the Lambeth convention criteria with more rigorous modifications. The ECG lead II was recorded using Power Lab 4/25T (AD Instruments, Inc., Colorado Springs, CO, USA) to determine the incidence and duration of VF.

### Heart rate variability (HRV) analysis

HRV was used as an indirect measurement of autonomic balance of the heart [53]. HRV analysis was performed in 10 min intervals obtained from ECG data collected throughout the entire myocardial I/R period. HRV was analyzed in in frequency-domain and time-domain using LabChart 8.0 software. In time-domain parameters, RMSSD are associated with parasympathetic output. Frequency-domain HRV parameters included the low frequency (LF) component, detected in the range of 0.04–0.15 Hz, which can reflect both sympathetic and parasympathetic modulation of heart rate. Additionally, the high frequency (HF) component, detected in the range of 0.15–0.40 Hz, is primarily associated with parasympathetic modulation of heart rate. The ratio of LF to HF power can be employed as an index to gauge the balance between sympathetic and parasympathetic activity [54].

### Immunohistochemical staining

The hearts of rats were collected and then fixed in a 4% paraformaldehyde solution for 24 h, embedded in paraffin, and subsequently sectioned. After dewaxing and performing antigen retrieval, the sections were subjected to incubation with an IL-6 antibody (ab9324, Abcam, UK) and an NLRP3 antibody. (T55651, Abmart, China) overnight at 4 °C and then incubated with secondary antibody (A0277, Beyotime, China) for 1 h at 37 °C. View and capture images under a microscope.

### Elisa assay

Specific concentrations of serum cTnT, CK-MB, LDH, IL-6, TNF-α were measured by ELISA, using commercial Rat Cardiac Troponin T (cTnT) ELISA kit (CSB-E16443r, CUSABIO, China), Rat Creatine Kinase MB isoenzyme (CK-MB) ELISA kit (CSB-E14403r, CUSABIO, China), Rat L-Lactate Dehydrogenase (LDH) ELISA kit (CSB-E11324r, CUSABIO, China), Rat Interleukin 6 (IL-6) ELISA kit (CSB-E04640r, CUSABIO, China), Rat TNF-α ELISA kit (CSB-E11987r, CUSABIO, China), respectively. The levels of IL-1β and IL-18 in myocardium were detected according to Rat Interleukin 1β (IL-1β) ELISA Kit (CSB-E08055r, CUSABIO, China) and Rat Interleukin 18 (IL-18) ELISA Kit (DY521-05, R&D Systems, USA).

### Tunel staining

Tunel staining was performed using a Tunel detection kit (40307ES50, Yeasen, China). After deparaffinization and rehydration, Paraffin sections were incubated in equilibration buffer for 10 min, followed by working strength TdT enzyme at 37 °C for 1 h. Pyroptotic cells were then labeled with fluorescein using Alexa Fluor 488-12-dUTP Labeling Mix. Nuclei were then subjected to staining using DAPI. The percentage of pyroptotic cell was determined by dividing the total count of Tunel-positive nuclei by the overall count of nuclei stained with DAPI in 5 fields for each animal. Subsequently, the images were analyszed utilizing a fluorescent microscope (Zeiss, Germany) and Image J software (NIH).

### Ultrastructural examination of myocardium

Fresh myocardium samples were meticulously sectioned into 1 mm^3 fragments. These fragments were sequentially treated with 4% glutaraldehyde and 1% osmic acid for immobilization. Following immobilization, the samples underwent a dehydration process using acetone. Subsequently, the samples were embedded in Epon812 resin, stained with toluidine blue, and sectioned into 70 nm slices. To enhance contrast and visualization, the slices were double stained with uranyl acetate and lead citrate. The transmission electron microscope (FEI-Tecnai G2) was then employed to examine the ultrastructure of myocardium. Visible images were captured at magnifications of 6000×.

#### AAV9-cTNT-OGDHL treatment

Recombinant adeno-associated virus serotype 9 (AAV9) vectors carrying the OGDHL or a negative control under the control of a c-TNT promoter (referred to as AAV9-cTNT-OGDHL and AAV9-ctrl, respectively) were produced by Genechem Co., Ltd (Shanghai, China). Rats were administered AAV9-cTNT-OGDHL and rAAV9-cTNT-ctrl at a dose of 3 × 10^11^ vector genomes (vg) per rat via intravenous tail injection. After a period of 3 weeks, the rat underwent either I/R surgery or sham surgery as per the experimental design.

### Cell cluture and treatment

Neonatal rat cardiac ventricular myocytes (NRCMs) were isolated from 1 to 3d old Sprague–Dawley rats as described previously [55]. NRCMs were cultured in DMEM (Gibco, USA) with 5% FBS (Gibco, USA), 10% HS (Gibco, USA) at 37 °C in a 5% CO_2_ incubator. To establish a hypoxia/reoxygenation (H/R) model, NRCMs were exposed to a hypoxia environment (1%O_2_, 5%CO_2_, 94% N_2_) in the presence of glucose-free DMEM medium for 6 h. For reoxygenation simulation, NRCMs were transferred back to a normoxic incubator and incubated in normal-glucose DMEM for 4 h. As required by experiment, Carbachol (an agonist for both nAChR and mAChR, HY-120,620, MCE, 0.5 × 10 M) was administered with or without Otenzepad (an M_2_ receptor antagonist, HY-101,381, MCE, 0.5 × 10 M) from the onset of H/R. NRCMs were transfected with Ad-OGDHL or Ad-ctrl for 24 h, then follow-up experiments.

### ATP assay

NRCMs were divided into four groups and seeded onto a 96-well plate respectively. After treatments, the cells were washed with cold PBS. The ATP assay Kit (ab83355, Abcam, UK) was used to measure levels of ATP in the cell lysates by colorimetric assay according to the manufacturer’s instructions. The resultss were subsequently adjusted based on the protein concentrations of each respective test.

### Immunofluorescent staining

NRCMs were subjected to postfixation in 4% paraformaldehyde for 25 min at room temperature, then treatment with 0.2% Triton X-100 for 20 min. Then NRCMs were incubated in PBS containing 10% goat serum for 1 h and exposed to Cleaved GSDMD-N antibody (ab215203, Abcam, USA) at a dilution of 1:200, allowing for an overnight incubation at 4℃. Subsequently, the cells were incubated with a secondary antibody conjugated with fluorescein isothiocyanate (FITC) for 2 h at room temperature. Simultaneously, the nucleus was stained with DAPI at a concentration of 100 ng/ml for a duration of 5 min. Zeiss fluorescence microscope (Carl Zeiss) was used to visualize the GSDMD-N terminal fluorescence, especially whether GSDMD-N protein translocated from cytosol to plasma membrane.

### mtROS detection in NRCMs

The measurement of ROS levels was conducted using the MitoSOX™ Red reagent (Invitrogen, China) following the manufacturer’s instructions. To perform the assay, Neonatal rat cardiac myocytes were treated with MitoSOX™ at a concentration of 5 μM for a duration of 10 min at a temperature of 37 °C. Then NRCMs were thoroughly washed three times to remove any residual reagent. Subsequently, the fluorescent intensity was assessed utilizing a confocal microscope (AXIO, Zeiss, Germany).

### Adenoviruses construction and cell infection

To achieve OGDHL overexpression, Ad-OGDHL or Ad-ctrl was generated according to the manufacturer’s protocol (Genechem Technology, China). NRCMs were infected with Ad-OGDHL at a multiplicity of infection of 50, as well as Ad-ctrl as a negative control. Western blot analyses were performed to determine the efficiency of gene infection 24 h after transfection.

### OGDHL activity

OGDHL activity in OGDH complex was represented by measurement of the alpha-ketoglutarate dehydrogenase activity based on NADH production using a colorimetric assay kit (ab185440, Abcam, USA) according to the manufacturer’s instruction.

### Western blot

Protein extraction was performed as follows. 200–250 g heart tissue was ground in an 1ml tube containing five 3-mm-diameter zirconia beads (G0203-150G, Servicebio, China) using a cryogenic grinding machine (KZ-III-F, Servicebio). After removing zirconia beads from the tube, tissue protein was lysed with lysis buffer supplemented with protease inhibitors (P0013, Beyotime, China) on ice for 20 min and then centrifuged at 18,000 x g for 30 min at 4 °C. Protein concentrations were determined with the BCA Protein Assay (23,225, Thermo, USA). The first antibody incubation took place overnight at a temperature of 4 °C using the following antibody concentrations: NLRP3 (T55651, Abmart, 1:1000), Total and cleaved N-terminal GSDMD (P30823, Abmart, 1:1000), Caspase-1 and cleaved Caspase-1 (WL03325, Wanleibio, 1:1000), OGDHL (17110-1-AP, proteinte, 1:1000) and GAPDH (14C10, CST, 1:1000). Protein levels were quantified using ImageJ software and normalized to GADPH.

### Data analysis

Continuous variables and categorical variables were described as mean ± SEM and percentages, respectively. One-way Analysis of variance (ANOVA) followed by Tukey’s correction was used for the comparison of three groups. VF incidence was analyzed using a chi-squared test. All statistical tests were performed using GraphPad Prism software version 9.0. P < 0.05 was considered statistically significant (* indicate P < 0.05, * * indicate P < 0.01, * * * indicate P < 0.001, and **** indicate P < 0.0001). NS means not significant between groups.

### Electronic supplementary material

Below is the link to the electronic supplementary material.

Supplementary Material 1

## Data Availability

The datasets generated or analysed in this study can be obtained upon reasonable request from the corresponding author.

## List of abbreviations

α7nAChR: Nicotinic acetylcholine receptor
AAV9: Adeno-associated virus serotype 9
ACh: Acetylcholine
Ad: Adenoviruses
CAP: Cholinergic anti-inflammatory pathway
eVNS: Electrical vagus nerve stimulation
I/R: Ischemia-reperfusion
M_2_AChR: M_2_ACh receptor
mAChRs: Muscarinic receptors
mtROS: Mitochondrial reactive oxygen species
mVNS: Magnetic vagus nerve stimulation
nAChRs: Nicotinic receptors
NRCMs: Neonatal rat cardiomyocytes
OGDHL: 2-oxoglutarate dehydrogenase like
PES: Programmed electrical stimulation
ROS: Reactive oxygen species
SPIO: Superparamagnetic iron oxide
VF: Ventricular fibrillation
