# Supplementary material for: Magnetic vagus nerve stimulation alleviates myocardial ischemia-reperfusion injury by the inhibition of pyroptosis through the M2AChR/OGDHL/ROS axis in rats
Source: J Nanobiotechnology. 2023 Nov 14;21:421. doi: 10.1186/s12951-023-02189-3 (PMC10644528; doi:10.1186/s12951-023-02189-3)
Supplement: Supplementary file 1 — Supplementary Material 1 [file 12951_2023_2189_MOESM1_ESM.docx]

**Supplemental Figures and Figure Legends**


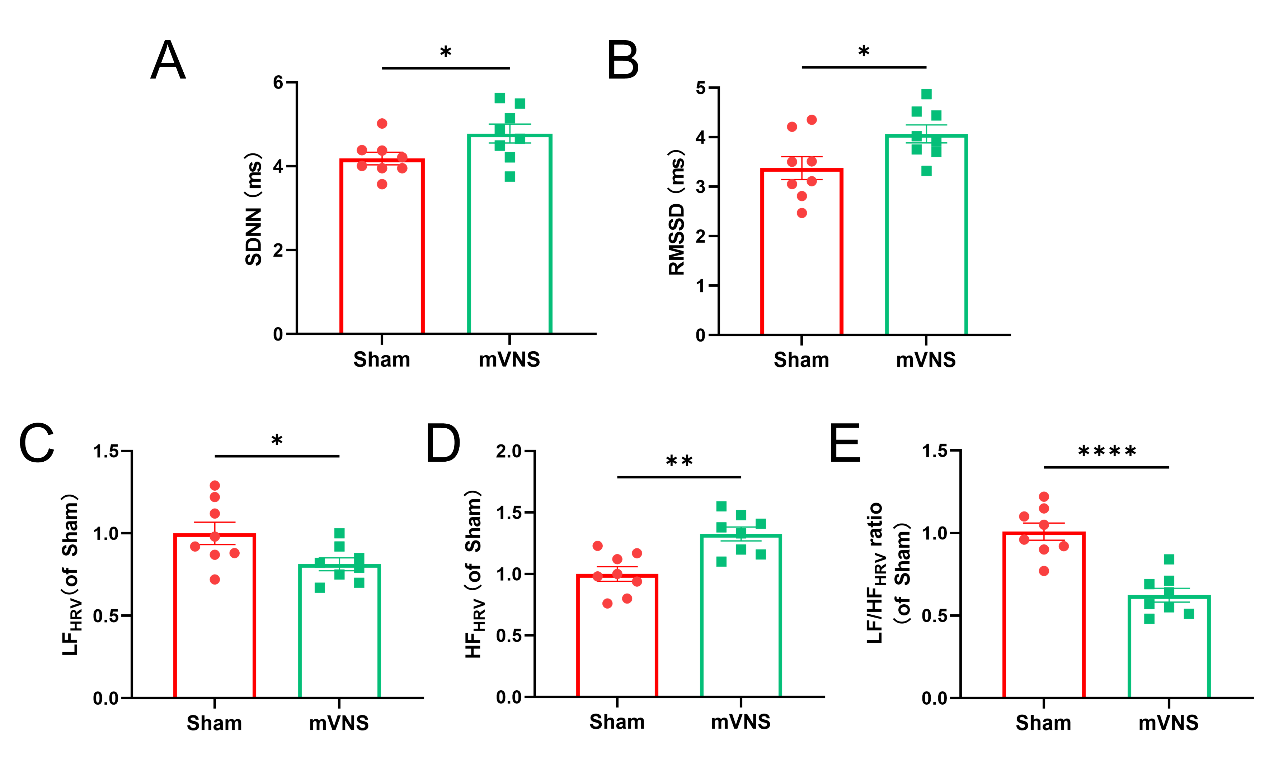


**Figure S1. Effect of mVNS on heart rate variability (HRV) alterations in rats (n=8 per group).** (A-B) The response of autonomic function measured by HRV in Sham or mVNS conditions. In mVNS group, the measurements of SDNN (A) and RMSSD (B) were significantly higher than those in Sham group. (C–E) Summary of the effects of mVNS on (C) low frequency (LF) component of the HRV, (D) high frequency (HF) component of HRV and (E) LF/HF ratio. mVNS displayed a decrease of LF component, an increase of HF component and consequently a decrease of LF/HF ratio. Data are mean ± SEM. *P < 0.05, **P < 0.01, ****P < 0.0001; All P values were obtained by Student’s t-test.
